# Bedside handover training and its effects on nurses’ knowledge and compliance

**DOI:** 10.1186/s12912-025-04075-9

**Published:** 2025-11-26

**Authors:** Warda Elbastawisy Ahmed, Samah Faisal Fakhry, Fawzia Mohamed Mohamed Badran

**Affiliations:** Faculty of Nursing, Ain Shams University, Cairo, Egypt

**Keywords:** Bedside handover, ISBAR, Nursing compliance, Patient safety, Communication, Clinical training

## Abstract

**Background:**

Effective communication during nursing handover is vital to ensure patient safety and continuity of care. Bedside handover, where patient information is communicated in the presence of the patient at the bedside, has gained significant attention in contemporary nursing practice.

**Aim:**

To evaluate the effect of bedside handover training on nurses’ related knowledge and compliance.

**Methods:**

A quasiexperimental, one-group pre-postfollow-up design was implemented in six intensive care units (ICUs) at General Mahalla Hospital. The study sample included 18 head nurses and a stratified random sample of 162 staff nurses affiliated with the Egyptian Ministry of Health. Data collection tools included a Nurses’ Knowledge Questionnaire, an observation checklist for bedside handover practice, and an audit sheet for handover reports. Data were collected at three time points: before training, immediately after, and at a 3-month follow-up.

**Results:**

“Significant improvements were observed across all measured outcomes for both head and staff nurses following the intervention (*p* < 0.001). Head nurses’ knowledge scores increased from 12.4 ± 3.7 to 89.5 ± 4.4 posttraining and 90.6 ± 3.2 at follow-up. Staff nurses similarly improved from 10.6 ± 4.2 to 89.6 ± 5.7 and 85.5 ± 6.4. ISBAR scores rose markedly in both groups (head: 25.7 ± 6.5 to 77.8 ± 5.8; staff: 21.1 ± 7.0 to 82.6 ± 6.6), with sustained although slightly reduced performance at follow-up. Audit compliance scores also improved significantly, with all gains statistically sustained at follow-up (*p* < 0.001). Strong positive correlations were found between knowledge, ISBAR practice, and audit scores, and regression analysis confirmed the intervention as the strongest predictor of compliance.

**Conclusion:**

Structured bedside handover training effectively enhances nurses’ communication skills and compliance with standardized handover practices, contributing to improved patient safety. Healthcare institutions should adopt structured training, reinforce ISBAR utilization, and conduct regular audits to sustain quality improvements in handover practices.

**Trial registration number:**

Not applicable.

**Clinical trial number:**

Not applicable.

## Introduction

Effective communication during patient handover is a cornerstone of safe and high-quality healthcare delivery [1]. Among various handover methods, the bedside handover process, wherein the exchange of clinical information occurs at the patient’s bedside, has gained increasing attention because of its potential to increase patient safety, improve nurse accountability, and foster patient involvement [2]. Despite its advantages, consistent and compliant implementation remains a challenge, often owing to gaps in nurses’ knowledge and understanding of proper handover techniques. Bedside handover training is proposed as a strategic intervention to bridge this gap and reinforce standard practices [3].

The nursing profession operates in an environment that demands continuous communication, particularly during shift transitions. Errors during handovers are a well-documented cause of adverse patient outcomes, and ineffective communication has been linked to sentinel events globally Murigi et al. [4]. In response, healthcare organizations have been urged to standardize handover processes to mitigate risks. Bedside handover represents not only a communicative function but also an opportunity to involve patients in their care, verify information in real time, and improve care continuity Le et al. [5]. However, the success of this approach heavily relies on the nurse’s knowledge of the handover process and adherence to established protocols [6].

Bedside handover directly addresses this by encouraging real-time information exchange in the presence of the patient, ensuring that crucial clinical details are clarified, validated, and acted upon promptly. Moreover, bedside handovers reduce the risk of information being lost or misunderstood, particularly during shift transitions in high-turnover units such as intensive care or emergency departments [2]. This practice also enables immediate verification of patient identifiers, medications, and planned interventions, which strengthens adherence to safety protocols and supports early detection of clinical deterioration [7]. Despite its advantages, miscommunication remains a critical risk when bedside handover is improperly executed. Factors such as unclear language, lack of structure, omission of key information, and environmental distractions can lead to adverse events [8].

Unlike traditional handovers conducted away from the patient’s presence, bedside handovers aim to actively involve patients and their families. This inclusion is aligned with the principles of person-centered care, promoting better communication, trust, and engagement [9]. Studies have shown that patient involvement in handovers increases patient satisfaction with and understanding of care plans, which can enhance compliance with treatment and reduce anxiety [10].

International bodies such as the Joint Commission International (JCI) and the World Health Organization [11] emphasize structured handover communication in their patient safety guidelines. The identification, situation, background, assessment, and recommendation (ISBAR) model is widely recommended for use during handovers to standardize information transfer and reduce errors [12]. This model promotes clarity by guiding healthcare professionals to introduce themselves and the patient (identification), state the reason for handover (situation), provide relevant clinical history (background), present their assessment (assessment), and suggest appropriate next steps (recommendation) Pakcheshm et al. [13]. Despite this endorsement, inconsistent implementation remains a concern, especially in high-pressure settings such as critical care units. Numerous barriers hinder the effective application of bedside handover. These include time constraints, the fear of breaching patient privacy, a lack of staff training, and resistance to change. A systematic review revealed that while this practice improves communication and safety, challenges such as cognitive overload, unclear accountability, and environmental factors reduce its adoption [14].

Research on bedside handover has clinical and educational imperatives. From a clinical perspective, reducing handover errors can substantially decrease preventable adverse events, thus improving patient outcomes (Lee et al. [15]). From an educational standpoint, understanding the efficacy of training on handover skills can guide curriculum development and professional development programs (Chung et al. [16]). It should thus explore not only knowledge acquisition but also behavioral changes in clinical practice, critical factors in the successful integration of bedside handover into routine nursing care [17].

Moreover, research focusing on measurable outcomes, such as improvements in nurses’ knowledge and adherence to bedside handover protocols, can inform institutional policies and standard operating procedures [18]. If training is proven effective, it could serve as a model for ongoing professional development programs and be incorporated into orientation packages for new nursing staff. Furthermore, it could support accreditation standards and help healthcare institutions meet regulatory requirements related to patient safety and communication [19].

Additionally, there is a need for research evaluating the impact of structured bedside handover training on nurses’ knowledge and compliance. It provides empirical data on whether training programs can lead to measurable improvements in bedside handover practices Pun et al. [20]. These findings are crucial for hospital administrators, nurse educators, and policy makers striving to strengthen clinical governance and patient safety [21].

Further research is needed to improve patient safety and enhance nursing practices by helping identify critical gaps in the literature through providing evidence-based insights into the effectiveness of bedside handover training [22].

### Significance of the study

Patient safety is mostly related to ineffective communication among healthcare team members. This is often due to inadequate handover, leading to a lack of information or misinterpretation. These factors jeopardize patient safety, leading to poor patient outcomes. Bedside handover is critical in the transfer of information and assignment of responsibilities between nursing shifts. It has the advantages of involving patients and enhancing their participation in their care plans. However, a previous study by the researcher demonstrated clear inadequacy in the conduction of bedside handover in the study setting. Hence, it is anticipated that a training program can improve their practice of such a critical procedure in nursing care.

The main advantages of bedside handover are early assessment, direct visualization, and one-patient focus. With early assessment, incoming nurses are able to instantly perform a basic assessment of the patient, which would reduce the risk of patient complications from the end of the study. The nurse closely linked to early assessment can perform a preemptive assessment, thus reducing handover errors for discrepancies between what the nurse sees on the patient and what has been communicated [23, 24].

There is a well-documented risk of information omission during bedside handovers, which can compromise the continuity and safety of patient care. Drach-Zahavy et al. [25] reported that only 20% to 47% of the information communicated during handovers was retained by incoming nurses, indicating significant gaps in the transfer of critical clinical details. However, structured educational and training interventions have been shown to mitigate these risks. Khalaf et al. [26] emphasized that targeted training programs can enhance information retention and handover quality, thereby improving communication accuracy and reducing the likelihood of clinical errors.

However, maintaining these improvements over time requires reinforcement. Studies have shown that initial compliance may decline without continuous professional development and managerial support. A longitudinal study by Fahajan et al. [27] reported that while ISBAR training improved nurses’ communication and perceptions of safety, sustained outcomes were dependent on regular refreshers and supervision. Given these insights, evaluating the effectiveness of bedside handover training in structured environments such as critical care units is essential. The current literature highlights the need for ongoing training, standardized procedures, and supportive leadership to ensure consistent handover practices that enhance both nurse performance and patient safety [28].

Despite the growing body of evidence supporting the value of structured communication tools such as ISBAR, implementation remains inconsistent across healthcare settings, particularly in critical care units (CCMs) Reime et al. [29]. Many nurses continue to rely on informal or unstructured methods of information transfer, which can undermine the quality and safety of care. Furthermore, there is limited research examining the long-term impact and sustainability of bedside handover training in high-acuity settings, especially in resource-constrained environments. This underscores the need for studies that not only assess immediate improvements but also explore retention of knowledge and practice over time. Therefore, the current study aims to evaluate the effectiveness of a structured bedside handover training program on nurses’ knowledge, ISBAR communication practices, and documentation compliance within intensive care settings [30].

## Aim of the study

The aim of this study was to evaluate the effects of bedside handover training on nurses’ related knowledge and compliance.

This aim was achieved through the following objectives:

1. Assessing nurses’ knowledge of and compliance with bedside handover before training.
2. Training nurses in bedside handover.
3. Evaluating nurses’ knowledge of and compliance with bedside handover after training.

## Research hypotheses

### H1

Bedside handover training improves nurses’ related knowledge.

### H2

Bedside handover training improves nurses’ compliance.

## Operational definition

### Nurses’ compliance

This refers to the degree to which staff nurses adhere to the standardized bedside handover protocol, including the consistent and accurate application of the ISBAR framework (Introduction, Situation, Background, Assessment, Recommendation) during patient handovers. In this study, compliance is assessed through an observational audit tool developed by the researcher, which evaluates the presence and quality of key handover elements. Higher compliance scores indicate better adherence to safe and effective handover practices.

#### Measurement

Measured via an observational audit checklist that is based on ISBAR elements. The tool includes 13 key indicators rated as “Done” or “Not Done” during real-time handover observations. The scores are totaled and expressed as percentages. Higher scores indicate greater compliance with standard handover procedures. Interrater reliability was established through pilot observations.

## Methods

### Design

A quasiexperimental one-group research design with pre-post and follow-up *assessments* will be used to conduct the study.

### Setting

The study was conducted *at* General Mahalla Hospital, one of the largest and most prominent public hospitals in the Nile Delta region. With a capacity of 215 beds, the hospital serves as a vital hub for specialized medical care, education, and clinical training. It includes six critical care units: *the medical* ICU, *surgical* ICU, *pediatric* ICU, *neurosurgical* ICU, *neonatal* ICU, and *cardiac care unit* (CCU). These units are equipped with advanced monitoring and life-support systems, enabling the delivery of complex and high-acuity care. *In general,* Mahalla Hospital plays a pivotal role in the region’s healthcare system, providing comprehensive services to diverse patient *populations*. Its strong administrative support, experienced nursing leadership, and emphasis on continuous professional development *make* it an ideal setting for implementing and evaluating bedside handover training. The inclusion of various critical care specialties offered a dynamic and multifaceted environment, enriching the study’s relevance and enhancing the generalizability of its findings across intensive care contexts. The hospital consists of two separate buildings. The first 5-floor building includes all *the* inpatient services. The ground floor is for hospital administration, *the* information center, medical *emergencies, the* radiology laboratory, *the* blood bank, Laundromat, sterilization, and the kitchen. The first floor has the dialysis and economic departments, *the* nursing administration office, and the quality department. The second *category includes* neonate ICUS, operation rooms, and gynecology and pediatrics departments. The third *facility* hosts four ICUs and female surgical and orthopedics wards. The fourth *ward included* male general surgery, *neurosurgery*, and *medical* and *orthopedic* wards. The fifth hosts *are* doctors and nurses. It provides free and economic services for all patients.

### Sampling

The study subjects consisted of all head/assistant head nurses and staff nurses working in the study settings during the time of the study. A total of 371 staff nurses and 18 head and assistant head nurses were included. The study included two different samples or groups: head nurses and staff nurses. These samples included nurses who were working in the previously mentioned settings at the time of the study, regardless of their age, gender, qualifications, or years of experience. The sample size for head/assistant head nurses was 18, and all of them were included in the study. For staff nurses, the sample size was calculated to detect an improvement in the scores of compliance with bedside handovers, with a moderate effect size (0.35) according to Brydges [31]. Using the G*Power software package, Version 3.1.9.4 [32], at a 95% confidence level and 80% power, the required sample size was 130. Taking into account a dropout rate of approximately 10%, the sample size was increased to 162. Thus, 9 staff nurses were selected for each head nurse. A stratified random sampling technique was used for selecting staff nurses, with head nurses/units used as strata so that each head nurse had 9 staff nurses selected. All head/assistant head nurses were included. Participants were selected on the basis of the following inclusion criteria: registered head nurses, staff nurses, and assistant head nurses working in critical care units with a minimum of two years of clinical experience in their respective roles and willingness to voluntarily participate in the study with the ability to attend all bedside handover training sessions and complete all pre, post, and follow-up assessments as needed. The exclusion criteria included nurses who had previously received formal training or participated in structured programs related to bedside handover or communication frameworks (e.g., ISBAR) to eliminate the influence of prior exposure on the study outcomes, staff on extended leave or rotation during the data collection period, or those with scheduling conflicts that prevented full participation in the intervention.

### Data collection tools

The data for the study were collected via three tools: a predesigned questionnaire, an observation checklist, and an audit sheet for handover reporting.

## Tool I: nurses’ knowledge questionnaire about bedside handover

“The tool was developed by the researcher on the basis of relevant literature [33–36], and its validity and reliability were established prior to use. It was administered both before and after the intervention and consisted of two main parts:”

“The tool was developed by the researcher on the basis of relevant literature [33–36], and its validity and reliability were established prior to use. It was administered both before and after the intervention and consisted of two main parts:”

Part I: Nurses’ personal characteristics included age, sex, nursing qualification, years of experience, marital status, residence, family income, and attendance of any course in administration or handover.

Part II: Nurses’ knowledge regarding bedside handover (pre/post and follow-up). It is used to assess head and staff nurses’ bedside knowledge. It consists of (41) questions; (multiple choice) questions, which are also classified into nine dimensions: concept/importance (4 questions); role of the nurse (3 questions); element (5 questions); importance of nurses’ compliance (6 questions); handover and patient safety and security (4 questions); the ISBAR concept/components (7 questions); ISBAR advantages (3 questions); challenges (6 questions); and improving nurses’ compliance (6 questions).

Part II: Nurses’ knowledge regarding bedside handover (pre/post and follow-up). It is used to assess head and staff nurses’ bedside knowledge. It consists of (41) questions; (multiple choice) questions, which are also classified into nine dimensions: concept/importance (4 questions); role of the nurse (3 questions); element (5 questions); importance of nurses’ compliance (6 questions); handover and patient safety and security (4 questions); the ISBAR concept/components (7 questions); ISBAR advantages (3 questions); challenges (6 questions); and improving nurses’ compliance (6 questions).

Scoring system: For each knowledge item, a correct response was given a score of 1, and an incorrect response was given a score of zero. The total grade was 41, the scores of the items were summed, and the total score was divided by the number of items, resulting in a mean score for each item. Means, standard deviations and medians were computed for quantitative analyses. For categorical analyses, the scores were converted into percentage scores. Knowledge was considered satisfactory if the percentage score was 60% or more and unsatisfactory if it was less than 60% [36].

## Tool II: nurses’ observation checklist regarding bedside handover practice

This scale was developed by Bakon et al. [37] and adopted from Ahmed [38] to assess the actual practices of head and staff nurses during bedside handover procedures before and after and during the follow-up phase after intervention. The various steps to be practiced by nurses during the handover procedure were covered. Each item observed was checked as “done”, “not done”, or “not applicable.” Two parts will be included.

Part I included nurses’ demographic data such as age, sex, nursing qualifications, years of experience, marital status, residence, family income, and attendance of any courses in administration or handover, in addition to identification data such as code number, unit, and time of observation.

Part II: This ISBAR tool “Identification” “situation, “background “assessment” “recommendation”. It was filled by observing nurses’ bedside handover practices. The tool included 13 statements covering the handover procedure under 5 dimensions. These include identification (2 items), situation (3 items), background (3 items), assessment (3 items), and recommendation (2 items). Each item was checked as “done/not done/not applicable for Bedside observation. Scoring: In the observation checklists, the items “not done” and “done” were scored “0” and “1”, respectively. The items “not applicable” were not scored and were discounted from the totals. For each part, the scores of the items were summed, and the total score was divided by the number of items, resulting in a mean score for the part. Means, standard deviations and medians were computed for quantitative analyses. For categorical analyses, the scores were converted into percentage scores. The practice was considered adequate if the percent score was 60% or more and inadequate if it was less than 60% [38].

Part II: This ISBAR tool “Identification” “situation, “background “assessment” “recommendation”. It was filled by observing nurses’ bedside handover practices. The tool included 13 statements covering the handover procedure under 5 dimensions. These include identification (2 items), situation (3 items), background (3 items), assessment (3 items), and recommendation (2 items). Each item was checked as “done/not done/not applicable for Bedside observation. Scoring: In the observation checklists, the items “not done” and “done” were scored “0” and “1”, respectively. The items “not applicable” were not scored and were discounted from the totals. For each part, the scores of the items were summed, and the total score was divided by the number of items, resulting in a mean score for the part. Means, standard deviations and medians were computed for quantitative analyses. For categorical analyses, the scores were converted into percentage scores. The practice was considered adequate if the percent score was 60% or more and inadequate if it was less than 60% [38].

## Tool III. Audit sheet for the handover report.

This tool was developed by researchers based on Street et al. [39]. It is used to evaluate the quality of bedside handover through a review of content and criteria, as documented by staff nurses and head nurses. Each item was checked as either “Documented,” “Not Documented,” or “Not Applicable.” It was used before and after the intervention.

Scoring system: The items checked “done” or “documented” were scored “1”, and the “not done” or “not documented” were scored “0.” The items “not applicable” were not scored and were discounted from the totals. The scores of the items of each dimension and for the total scale were summed, and the total score was divided by the number of corresponding items, yielding mean scores. Means, standard deviations and medians were computed for quantitative analyses. For categorical analyses, the scores were converted into percentage scores. An audit was considered adequate if the percentage score was 60% or greater and inadequate if it was less than 60% [38].

### Validity and reliability

The content and face validity of the instruments were assessed by a jury panel comprising six experts, including two professors and four assistant professors from the Nursing Administration Department and Surgical Nursing Department, Faculty of Nursing, Ain Shams University, Cairo, Egypt. The instruments were evaluated for their applicability, comprehensiveness, and relevance to the study objectives. The experts were invited to review the proposed instruments and provide their feedback. On the basis of their recommendations, necessary modifications were made, including the addition and exclusion of specific items to increase the clarity and appropriateness of the tools. We assessed the inter-rater reliability of the observation checklist used to evaluate nurses’ bedside handover practices; Cohen’s Kappa coefficient was calculated. The overall observation score was 0.90, indicating a high level of adherence to the criteria. Furthermore, the Kappa value for inter-rater agreement was 0.90, which reflects almost perfect agreement. This high Kappa value supports the consistency of the observations made by different raters and adds credibility to the observational data collected in the study.

The reliability of the data collection tools was assessed for internal consistency via the Guttman split-half Coefficient test. The nurses’ knowledge score for bedside handover was 0.98, the nurses’ observation score for bedside handover practice was 0.90, and the score on the Audit sheet for handover report was 0.96.

### Ethical considerations

We confirm that our study was conducted in accordance with the principles outlined in the Declaration of Helsinki. The Scientific Research Ethical Committee of the Faculty of Nursing, Ain Shams University, granted ethical permission under (code number: NUR 25.05.700). The Faculty of Nursing submitted official letters to the designated hospital to obtain permission for data collection. Following a thorough explanation of the investigation’s objectives and procedures, each participant (head/assistant nurses and staff nurses) provided informed written consent. The right of all the participants to refuse or withdraw from the study at any time was ensured. The complete confidentiality and anonymity of any information that was obtained were ensured. No actual or possible harm has been predicted from the investigation’s maneuvers.

## Pilot study

The pilot study was conducted on eighteen nurses, who made up 10% of the main study sample. It was used to test the clarity of the tools and the applicability of the study. It also served to estimate the time needed to conduct the interviews. On the basis of the results of the pilot study, modifications should be made. The pilot sample was not included in the main sample to avoid contamination since the study type was intervention.

## Field work

The actual fieldwork of the study extended over a period of seven months and involved several key phases, including preparation, implementation of the bedside handover training program, data collection, and follow-up. Each phase was carefully structured to ensure smooth integration of the intervention into the clinical setting and to maintain the reliability of the data collected.

### Preparation phase

Before the intervention commenced, comprehensive orientation sessions were conducted with head nurses and staff nurses working in the selected intensive care units. These sessions introduced the objectives, procedures, and expectations of the study and emphasized the importance of bedside handover in enhancing communication and patient safety. Informed consent was obtained from all participants, ensuring confidentiality and voluntary participation. The training program comprised seven sessions with a total contact time of 10.5 hours. A variety of teaching aids were employed, including PowerPoint slides, printed handouts, case scenarios, and bedside demonstration videos. The participants included 18 head and assistant head nurses, as well as 162 staff nurses drawn from six intensive care units (ICUs). The training was delivered by the primary researchers under the supervision of faculty mentors. To evaluate the effectiveness of the intervention, assessments were conducted at three points: before the training (pre-intervention), immediately after the training (post-intervention), and three months later (follow-up). These assessments utilized a knowledge questionnaire, the ISBAR observation checklist, and a structured handover audit sheet. Coordination with hospital administration and unit managers was undertaken to schedule training sessions and data collection in a way that minimized disruption to patient care as illustrated in Table 1.

**Table 1:** Training program schedule for bedside handover intervention

Session No.	Title/Focus Area	Core Content and Learning Objectives	Teaching & Learning Methods	Duration
Session 1	Introduction to Bedside Handover	Definition, purpose, and importance of bedside handover in patient safety; overview of communication failures and consequences.	Interactive lecture, open discussion.	90 min
Session 2	Role of Nurses in Quality and Safety	Nurses’ role in ensuring safe, effective communication; accountability and teamwork principles; link between handover and IPSG goals.	Group discussion, real-case reflection.	90 min
Session 3	Elements of the Handover Process	Components of complete and effective handover; timing, sequence, environment, and ethical considerations (confidentiality, privacy).	Demonstration and peer observation.	90 min
Session 4	ISBAR Framework: Concept and Application	Introduction and breakdown of ISBAR (Identification, Situation, Background, Assessment, Recommendation); rationale and evidence for structured communication.	Lecture, PowerPoint presentation, guided discussion.	90 min
Session 5	Practical Application of ISBAR in Critical Care	Applying ISBAR to ICU patient scenarios; verbal and written handover using simulated cases; handling interruptions.	Simulation practice, role-play exercises.	90 min
Session 6	Challenges and Barriers in Bedside Handover	Common implementation barriers (time pressure, environment, resistance to change, privacy); strategies to overcome them.	Brainstorming, case-based discussion.	90 min
Session 7	Improving Compliance and Sustainability	Reinforcement of knowledge; auditing techniques; continuous quality improvement; recap and feedback session.	Group presentation, reflective discussion, evaluation.	90 min

## Implementation of the bedside handover training program

The core component of the field work involved the implementation of a structured bedside handover training program for both head nurses and staff nurses. This program was designed around the principles of the ISBAR framework and delivered through multiple interactive training sessions. These sessions addressed the concept and importance of bedside handover, the role of nurses in ensuring patient safety, the essential elements of the handover process, and strategies to overcome common challenges in clinical practice.

The training was conducted via a combination of lectures, group discussions, case-based learning, and role-playing exercises, with the aim of strengthening both the theoretical understanding and practical application of bedside handover skills. Each session integrated clinical scenarios to ensure that nurses could confidently apply ISBAR principles in real-life situations.

Each training session was facilitated by experienced nurse educators specializing in clinical communication and patient safety. The sessions were delivered over a structured period of seven weeks, with weekly meetings lasting approximately ninety minutes each. The content was tailored to address the specific challenges of intensive care settings. The participants were encouraged to engage actively, share experiences, and practice bedside handover scenarios, thereby reinforcing knowledge and compliance with standardized procedures.

### Data collection

Data collection was conducted in two main phases: preintervention and postintervention.

## Preintervention data collection

Prior to the training program, baseline data were collected from all participants. The Nurses’ Knowledge Questionnaire was used to assess their understanding of bedside handover and the ISBAR framework. In addition, bedside handover practices were evaluated via an observational checklist, and documentation compliance was assessed via the audit tool. These instruments provide a comprehensive baseline assessment of knowledge, practice, and compliance.

## Post-intervention data collection

Following completion of the bedside handover training program, the same instruments were administered to all participants to measure changes in knowledge, ISBAR practice, and documentation compliance. The postintervention data collection took place one month after the final training session, providing sufficient time for participants to apply and reflect on the skills and knowledge gained.

## Follow-up and feedback

Following postintervention data collection, a follow-up phase was conducted three months later. This phase aimed to assess the long-term impact of the bedside handover training program on nurses’ knowledge, handover practices, and documentation compliance. The same instruments used in the pre- and postintervention phases were applied to evaluate retention and the ongoing application of skills. This phase was essential for determining the sustainability of the program’s effects and provided insights into areas requiring continuous reinforcement and managerial support.

## Data analysis and reporting

The collected data were systematically analyzed to identify significant differences between the pre- and postintervention scores, as well as between different categories of participants. This analysis was essential for evaluating the effectiveness of the bedside handover training program in improving nurses’ knowledge, ISBAR-based communication practices, and documentation compliance. The findings were compiled into a comprehensive report that highlighted key outcomes, practical insights, and recommendations for strengthening bedside handover practices in intensive care settings.

The field work was instrumental in generating comprehensive and actionable data on the impact of the training program. This study provides valuable insights into the enhancement of nurses’ communication and handover competencies, as well as their contribution to improving patient safety and continuity of care. The structured and well-coordinated phases of the field work ensured the successful completion of the study and contributed significantly to advancing the understanding of effective bedside handover practices in nursing.

## Statistical design

Data entry and statistical analysis were performed via the SPSS ‎‎20.0 statistical ‎software package. Quality control was performed at the coding and data entry stages. Data ‎were presented via descriptive statistics ‎in the form of frequencies and percentages for ‎qualitative variables and ‎means and standard deviations for ‎quantitative variables. Cronbach’s alpha coefficient was ‎calculated to ‎assess the reliability of the developed tools through their ‎internal consistency. ‎Quantitative continuous data were compared via ‎one-way analysis of variance ‎‎‎(ANOVA) or the Kruskal‒Wallis test, which were used as appropriate. Spearman rank correlation was used for the ‎assessment of the ‎interrelationships between quantitative variables and ‎ranked variables. Statistical ‎significance was considered at a *p* value < 0.05.‎

## Results

### Demographic characteristics of head nurses (n = 18)

Table 2: The majority of head nurses (77.8%) were under 40 years old, with a mean age of 38.2 ± 1.9 years. All participants were female. Most held a master’s degree (94.4%), and 61.1% had more than 16 years of experience. In terms of marital status, 94.4% were married, and 61.1% resided in urban areas. A high percentage (83.3%) reported insufficient income. While 50% had taken courses in administration, only 5.6% had received prior handover training.

**Table 2:** Demographic characteristics of the head nurses in the study sample (*n* = 18)

	Frequency	Percent
Age:		
< 40	14	77.8
40+	4	22.2
Range	36.0–43.0	
Mean±SD	38.2±1.9	
Median	38.0	
Gender:		
Male		
Female	18	100.0
Nursing qualification:		
Master	17	94.4
Doctorate	1	5.6
Experience years:		
< 16	7	38.9
16+	11	61.1
Range	14.0–21.0	
Mean±SD	16.1±1.8	
Median	16.0	
Marital status:		
Unmarried	1	5.6
Married	17	94.4
Residence:		
Rural	7	38.9
Urban	11	61.1
Family income:		
Insufficient	15	83.3
Sufficient	3	16.7
Had courses in administration:		
No	9	50.0
Yes	9	50.0
Had courses in handover:		
No	17	94.4
Yes	1	5.6

### Demographic characteristics of staff nurses (n = 162)

Table 3: The majority of staff nurses (80.9%) were over 25 years old, with a mean age of 25.5 ± 1.2 years. Females represented 94.4% of the sample. Almost all nurses (99.4%) held a bachelor’s degree or higher. Additionally, 99.4% had five or more years of experience, with a mean of 3.8 ± 1.2 years. In terms of marital status, 58% were married. Most participants lived in rural areas (59.3%) and reported insufficient income (92%). Notably, 99.4% had not taken courses in administration, and 88.9% had not received any training on handover.

**Table 3:** Demographic characteristics of staff nurses in the study sample (*n* = 162)

	Frequency	Percent
Age:		
< 25	31	19.1
25+	131	80.9
Range	23.0–28.0	
Mean±SD	25.5±1.2	
Median	26.0	
Gender:		
Male	9	5.6
Female	153	94.4
Nursing qualification:		
Diploma	1	0.6
Master	159	98.1
Doctorate	2	1.2
Nursing qualification:		
Diploma	1	0.6
Bachelor+	161	99.4
Experience years:		
< 5	1	0.6
5+	161	99.4
Range	1.0–8.0	
Mean±SD	3.8±1.2	
Median	4.0	
Marital status:		
Unmarried	68	42.0
Married	94	58.0
Residence:		
Rural	96	59.3
Urban	66	40.7
Family income:		
Insufficient	149	92.0
Sufficient	13	8.0
Had courses in administration:		
No	161	99.4
Yes	1	0.6
Had courses in handover:		
No	144	88.9
Yes	18	11.1

### Comparison of head and staff nurses’ knowledge, ISBAR practices and audits throughout the study phases

Table 4: The comparison between head and staff nurses revealed no significant difference in post intervention knowledge scores (*p* = 0.88). However, staff nurses scored significantly higher in both ISBAR and audit practice posttraining (*p* = 0.003 for both). At follow-up, head nurses retained significantly higher knowledge scores (*p* < 0.001), whereas no significant differences were observed for audit or ISBAR. These results suggest a generally comparable intervention effect, with some role-based variations.

**Table 4:** Comparison of head and staff nurses’ knowledge, ISBAR practices and audits throughout the study phases

	Group (Mean±SD)		T-test	p value
	Head (n = 18)	Staff (n = 162)		
PRE				
Knowledge	12.4±3.7	10.6±4.2	1.88	0.07
ISBAR	25.7±6.5	21.1±7.0	2.85	0.009*
Audit	28.7±6.4	27.7±6.3	0.60	0.55
POST				
Knowledge	89.5±4.4	89.6±5.7	−0.16	0.88
ISBAR	77.8±5.8	82.6±6.6	−3.30	0.003*
Audit	79.5±5.3	83.9±6.4	−3.32	0.003*
FU				
Knowledge	90.6±3.2	85.6±6.4	5.50	< 0.001*
ISBAR	71.8±5.9	74.8±5.3	−2.06	0.052
Audit	74.8±4.4	75.8±4.3	−0.91	0.37

(*) Statistically significant at p < 0.05

### Head and staff nurses’ knowledge, ISBAR practices and audits throughout the study phases

Table 5: Repeated-measures ANOVA revealed statistically significant improvements in knowledge, ISBAR, and audit scores from pre- to postintervention and at follow-up for both groups (*p* < 0.001). Head nurses demonstrated slightly greater knowledge retention, whereas staff nurses showed stronger ISBAR performance posttraining. These findings affirm the intervention’s effectiveness in enhancing critical aspects of handover communication.

**Table 5:** Head and staff nurses’ knowledge, ISBAR practices and audits throughout the study phases

	Time (Mean±SD)			F-test	p value
	Pre	Post	FU		
Head (*n* = 18)					
Knowledge	12.4±3.7	89.5±4.4	90.6±3.2	2463.36	< 0.001*
ISBAR	25.7±6.5	77.8±5.8	71.8±5.9	395.82	< 0.001*
Audit	28.7±6.4	79.5±5.3	74.8±4.4	483.79	< 0.001*
Staff (*n* = 162)					
Knowledge	10.6±4.2	89.6±5.7	85.5±6.4	10507.85	< 0.001*
ISBAR	21.1±7.0	82.6±6.6	74.8±5.3	4554.54	< 0.001*
Audit	27.8±6.3	83.9±6.1	75.8±4.3	4697.20	< 0.001*

(*) Statistically significant at p < 0.05

### Correlation matrix of the knowledge, ISBAR, and overall audit scores

Table 6: Strong and statistically significant correlations were observed among knowledge, ISBAR, and audit scores in both nurse groups. Among staff nurses, knowledge was highly correlated with ISBAR (*r* = 0.806, *p* < 0.01) and audits (*r* = 0.791, *p* < 0.01). For head nurses, similar strong associations were found (e.g., knowledge and audit: *r* = 0.741, *p* < 0.01). These results indicate that improving knowledge is likely to enhance both documentation and handover practice quality.

**Table 6:** Correlation matrix of the knowledge, isbar, and overall audit scores

	Spearman’s rank correlation coefficient		
	Knowledge	ISBAR	Audit
Head (*n* = 18)			
Knowledge	1.000		
ISBAR	0.682**	1.000	
Audit	0.741**	0.908**	1.000
Staff (*n* = 162)			
Knowledge	1.000		
ISBAR	0.806**	1.000	
Audit	0.791**	0.888**	1.000
Total sample (*n* = 180)			
Knowledge	1.000		
ISBAR	0.790**	1.000	
Audit	0.782**	0.890**	1.000

(**) Statistically significant at p < 0.01

### Correlations between nurses’ knowledge, ISBAR, and overall audit scores and their characteristics

Table 7: Among staff nurses, years of experience was significantly positively correlated with knowledge, ISBAR, and audit scores (*r* values ranging from 0.094 to 0.104, *p* < 0.05). These associations were weaker and not significant for head nurses. The findings suggest that greater experience may enhance handover performance among staff-level nurses, underscoring the importance of early skill development.

**Table 7:** Correlation between nurses’ knowledge, ISBAR, and overall audit scores and their characteristics

	Spearman’s rank correlation coefficient		
	Knowledge	ISBAR	Audit
Head (*n* = 18)			
Age	0.004	−0.042	−0.009
Qualification level	0.070	0.144	0.137
Experience years	0.003	−0.040	−0.023
Staff (*n* = 162)			
Age	0.094*	0.086	0.087
Qualification level	0.058	0.029	0.025
Experience years	0.094*	0.104*	0.095*
Total sample (*n* = 180)			
Age	0.114**	0.041	0.042
Qualification level	0.065	0.042	0.037
Experience years	0.113**	0.056	0.048

(*) Statistically significant at p < 0.05

(**) Statistically significant at p < 0.01

### Best-fitting multiple linear regression model for the audit practice score

Table 8: ISBAR practice (β = 0.48, *p* < 0.001) was the primary predictor of improved audit scores, followed by age, experience, and participation in handover courses. This underscores the interconnectedness between structured ISBAR use and the overall quality of the handover audit results.

**Table 8:** Best-fitting multiple linear regression model for the audit practice score

	Unstandardized Coefficients		Standardized Coefficients	t test	p value	95% Confidence Interval for B	
	B	Std. Error				Lower	Upper
Constant	23.35	11.79		1.980	0.048	0.16	46.53
Intervention	18.41	4.46	0.32	4.126	< 0.001	9.63	27.18
Age	−1.12	0.51	−0.16	−2.202	0.028	−2.13	−0.12
Experience years	1.05	0.53	0.14	1.999	0.046	0.02	2.08
Courses in handover	2.62	1.19	0.05	2.206	0.028	0.28	4.95
Knowledge score	0.10	0.06	0.15	1.751	0.081	−0.01	0.22
ISBAR score	0.44	0.04	0.48	9.871	< 0.001	0.35	0.53

r-square = 0.97

Model ANOVA: F = 1827.31, p < 0.001

Variables entered and excluded: gender, qualification, job category, experience, marital status, residence, income, department, training courses in administration

## Cohen’s d effect sizes for pre-post- and pre-follow-up comparisons among head and staff nurses across study domains

Table 9: Effect sizes, measured by Cohen’s d, which indicated exceptionally large values across all study domains for both head and staff nurses. All effect size values exceeded the conventional threshold for a very large effect ( > 0.8), with several values substantially higher, ranging from 8.39–22.61. Among head nurses, the greatest effect was observed in the knowledge domain (post = 18.97; prefollow-up = 22.61), followed by audit and ISBAR, both of which demonstrated sustained improvements postintervention. Staff nurses showed a similarly strong trend, with very large effects on knowledge (post = 15.78; prefollow-up = 13.84), and both the ISBAR and audit domains exceeded 8.5. These findings highlight the significant and enduring impact of bedside handover training interventions on nurses’ knowledge and compliance practices. The magnitude of these effect sizes reflects a substantial and meaningful change, supporting both the effectiveness and the sustainability of the implemented program.

**Table 9:** Cohen’s d effect sizes for pre-post- and pre-follow-up comparisons among head and staff nurses across study domains

	Cohen-d (Effect Size)	
	Pre-post	Pre-FU
Head nurses		
Knowledge	18.97	22.61
ISBAR	8.46	7.43
Audit	8.65	8.39
Staff nurses		
Knowledge	15.78	13.84
ISBAR	9.04	8.65
Audit	9.05	8.90

The magnitudes of the effects are extremely large, mostly above 2.0, indicating substantial improvements following the intervention.

## Discussion

Bedside handover involves the outgoing nurse providing a verbal and often written report to the incoming nurse at the patient’s bedside, allowing the patient to be directly involved in the discussion. This approach contrasts with traditional handovers that occur away from the patient, such as in staff rooms or nursing stations. The goals of bedside handover are to ensure accurate and up-to-date transfer of clinical information; improve continuity and quality of care; engage the patient and family in the care process; increase patient satisfaction and trust; and reduce medical errors and omissions [40].

This study assessed the effectiveness of a structured bedside handover training program on nurses’ knowledge, ISBAR practices, and documentation compliance. The results demonstrated statistically significant improvements across all measured dimensions and sustained retention at follow-up. These findings reinforce the relevance of structured communication training as a pivotal strategy in enhancing patient safety and nurse performance.

### Enhancement in knowledge following bedside handover training

This study demonstrated a statistically significant improvement in nurses’ knowledge scores following bedside handover training, supporting the hypothesis that structured education effectively enhances cognitive understanding of communication principles and the ISBAR framework. Both head and staff nurses benefited from the intervention, with head nurses exhibiting notably greater knowledge retention at follow-up. This sustained retention among head nurses may be attributed to their leadership roles, which involve ongoing engagement with communication processes and patient safety measures. Their stronger foundational knowledge of structured communication likely positioned them to apply the ISBAR framework more effectively in practice. Additionally, their responsibility for supervising staff and ensuring adherence to best practices may have further motivated them to internalize and consistently reinforce the training content. Moreover, the local context played a crucial role in supporting these gains; strong leadership, a culture emphasizing effective communication and patient safety, and established policies reinforced the importance of accurate handover and documentation. The close teamwork among nursing staff also facilitated shared learning and consistent use of the ISBAR method across all levels. Together, enhanced communication knowledge and a supportive clinical environment contributed to the sustained improvements observed in both head and staff nurses.

These results align with those of Lee et al. [15], who emphasized that structured training programs promote deeper conceptual understanding, enabling nurses to conduct handovers with increased clarity and confidence. Consistent reinforcement through practice and feedback has also been shown to significantly increase nurses’ handover literacy and procedural confidence. Moreover, Akcoban et al. [21] reported that nurses trained with the ISBAR framework achieved higher knowledge acquisition and performance scores, highlighting the value of structured training as a foundational intervention. The notable improvement among staff nurses supports findings by Pun et al. [20], who concluded that structured ISBAR training enhances handover accuracy; Gheisari et al. [40], who reported that applying an ISBAR-based clinical supervision model significantly improved nursing students’ clinical decision-making and self-efficacy, highlighting the cognitive gains from structured frameworks.

### Improvement in the application of ISBAR as a practical compliance measure

Post intervention, there was a significant improvement in ISBAR practice scores, with staff nurses demonstrating greater gains than head nurses did. The structured ISBAR format appeared to enhance nurses’ ability to accurately recall and sequence critical patient information, thereby aligning with established communication standards. From the researcher’s standpoint, the notable improvement in ISBAR practice scores following the intervention highlights the effectiveness of structured communication training in improving adherence to standardized handover procedures. Staff nurses showed more pronounced gains compared to head nurses, which could be attributed to their initially lower familiarity with the ISBAR framework, allowing for greater observable progress. The structured format of ISBAR likely assisted nurses in systematically organizing and recalling essential patient details, thereby enhancing the accuracy and consistency of clinical communication. These results emphasize the importance of focused educational programs in strengthening communication practices, particularly among nursing staff involved in direct patient care.

These findings corroborate those of Reime et al. [29], who reported statistically significant increases in communication accuracy and completeness following ISBAR-based training during shift reports. The mnemonic nature of ISBAR is particularly beneficial for junior staff or those lacking prior formal training. Similarly, Kaltoft et al. [12] reported substantial improvements in compliance with bedside ISBAR protocols in emergency unit settings, highlighting the tool’s broad applicability. Furthermore, Yun et al. [41] demonstrated that SBAR-based simulation training significantly improved communication accuracy and adherence among less experienced nurses, supporting the effectiveness of structured simulation interventions in enhancing practical compliance. Additionally, Pakcheshm et al. [13] demonstrated that ISBAR implementation in emergency departments significantly reduced communication errors and improved patient flow, indicating that the benefits of ISBAR training are not confined to a single clinical environment.

### Audit findings support the implementation of bedside handover training in clinical practice

The audit results demonstrated significant post training improvements in documentation compliance for both head and staff nurses. For the head nurses, the mean audit score increased from 28.7 ± 6.4 before the intervention to 79.5 ± 5.3 after the intervention, which was sustained at 74.8 ± 4.4 at the follow-up. Staff nurses achieved similar gains, increasing from 27.8 ± 6.3 to 83.9 ± 6.1 postintervention and maintaining 75.8 ± 4.3%. These results indicate that the intervention not only enhanced knowledge and ISBAR practices but also ensured consistent adherence to documentation standards over time. The improvements seen in the audit results may have been influenced by the local context in which the training took place. Support from nursing leadership and a strong focus on patient safety are likely to make it easier for staff to accept and apply the new approach to a new handover. The hospital’s existing policies and culture may have already encouraged good communication and documentation practices, which helped reinforce the training. Also, because the nursing teams were familiar with each other and worked closely, it may have been easier to adopt the ISBAR framework together. Involving both head and staff nurses in the training also helped create a shared understanding and consistent use of the new method. These local factors likely played a key role in the success of the intervention and should be considered when thinking about using similar training in other settings.

The observed improvements in audit scores among both head and staff nurses indicate that the training intervention effectively translated into enhanced real-world documentation and compliance behaviors. These results underscore the clinical value of structured bedside handover education, particularly in high-risk settings such as critical care. Similarly, Cruchinho et al. [42] highlighted that targeted training programs enhance bedside handover fidelity, resulting in improved documentation practices and reduced information loss. Given that documentation plays a crucial role in legal accountability and patient safety, these findings further emphasize the significance of the training program. Additionally, El-Sayed Ghonem and El-Husany [43] demonstrated that combining audit processes with educational interventions effectively improved adherence to communication standards, and an emergency-department study reported increased documentation of key handover items following the implementation of a structured handover model [44]. Together, these findings support coupling bedside handover education with ongoing audit cycles to sustain documentation quality and safety. Additionally, Tasew et al. [45] demonstrated that integrating pre- and postintervention auditing with structured education significantly improves compliance with clinical handover documentation.

### Correlational dynamics between knowledge, ISBAR, and audit scores

The findings of the present study revealed strong positive correlations between knowledge and practice indicators (ISBAR and audit) in both nurse groups. This finding suggests that improved theoretical understanding directly supports improved practical application, validating the interdependence of the cognitive and behavioral learning domains. The significant positive relationships between knowledge and both ISBAR practice and audit scores highlight the important connection between understanding communication principles and effectively applying them in practice. This indicates that enhancing nurses’ knowledge of structured communication aids in improving handover and documentation skills. Nonetheless, since correlation doesn’t imply causation, other factors like organizational culture, leadership support, and individual motivation likely influence how knowledge translates into behavior. The larger gains seen in head nurses suggest that combining knowledge with leadership roles and a supportive environment is key to sustaining changes in practice. These findings emphasize the importance of training that combines knowledge development with ongoing reinforcement.

These findings align with the model of integrated learning proposed by Wang et al. [46], who argued that cognitive acquisition is a precursor to behavioral execution in clinical settings, especially when supported by repetitive practice and structured feedback loops. Alizadeh et al. [44] also proposed a model in which knowledge retention serves as a cognitive foundation for skill acquisition in handover routines. Moreover, the strong link between knowledge and practice is supported by a recent meta-analysis showing that implementation strategies focused on education significantly enhance nurses’ theoretical understanding, skills, and clinical behavior [47]. Similarly, El-Sayed Ghonem and El-Husany [43] reported a moderate positive correlation between nurses’ SBAR knowledge and their observed handoff practices following training (*r* = 0.66, *p* < 0.001), reinforcing the knowledge-to-practice pathway that our data suggest. Additionally, Jaber et al. [48] reported that increased handover knowledge scores were positively and significantly correlated with higher quality documentation audit scores following bedside communication interventions, reinforcing the knowledge–practice linkage in critical care contexts.

### Influence of clinical experience on knowledge and compliance

The findings of the present study demonstrated that years of clinical experience were significantly associated with higher postintervention knowledge and practice scores among staff nurses but not among head nurses. This may reflect the increased frequency of direct care and bedside interaction among staff nurses, allowing them to immediately apply training concepts. From researchers’ point of view, more experienced nurses tend to have a solid foundation that facilitates learning new communication frameworks like ISBAR. Their extensive clinical exposure likely improves their capacity to comprehend, integrate, and apply the training effectively. On the other hand, the absence of this association among head nurses may be explained by the relatively uniform experience levels within this group or by the nature of their leadership roles, which focus more on supervision and policy adherence rather than direct clinical practice. This indicates that while clinical experience enhances learning for staff nurses, other factors may be more influential in determining outcomes for nurse leaders.

These observations are consistent with those of Chung et al. [16] reported that less experienced nurses showed the most dramatic posttraining improvements due to the greater marginal utility of new knowledge for them. Hence, early-career exposure to handover protocols is crucial. The high scores of head nurses may reflect their leadership roles and prior exposure to handover protocols [49]. According to Le et al. [5], bedside handover education programs that integrate simulation and reflective feedback result in more significant practice changes than lecture-only methods do, supporting the instructional approach used in this study. Similarly, Ghosh et al. [1] reported that nurses with more than five years of bedside practice demonstrated higher posttraining compliance with ISBAR protocols, indicating that experience can facilitate the integration of structured communication models. In addition, Murigi et al. [4] reported that clinical experience moderated the relationship between handover training and documentation compliance, with experienced nurses sustaining improvements for longer periods postintervention.

### Predictors of improved knowledge and ISBAR compliance

Regression analysis revealed that participation in previous training and the clinical role (staff vs. head nurse) were significant predictors of postintervention knowledge and ISBAR compliance. Interestingly, age showed a negative association, suggesting that younger nurses may be more adaptable to new communication protocols. The regression results show that previous training and the nurse’s clinical role are important factors in predicting knowledge and ISBAR compliance after the intervention. Nurses with prior training likely had a better understanding of structured communication, which helped them learn and apply the new protocol more effectively. Differences between staff and head nurses may be due to their distinct responsibilities, with staff nurses possibly engaging more directly in handovers, reinforcing their compliance. The negative link with age suggests younger nurses might be more flexible and willing to adopt new communication methods, possibly because of recent education or greater openness to change. This underscores the importance of customizing training to address differences in experience and adaptability across age groups.

This finding aligns with that of Muller et al. [28], who reported that digital-native nurses tend to be more responsive to structured interventions. Tailoring training content to accommodate generational learning styles could further optimize outcomes. Moreover, the observed negative association between age and ISBAR compliance highlights the importance of customizing future training programs to address diverse learning needs across different age groups [11]. Husna et al. [50] also demonstrated that structured training and strict adherence to ISBAR protocols significantly improve documentation quality. Additionally, Gheisari et al. [40] demonstrated that an ISBAR-based clinical supervision model significantly enhanced nursing students’ self-efficacy and decision-making, aligning with our findings that structured communication interventions directly improve audit performance and that structured handover proficiency directly correlates with improved practice outcomes. Similarly, Gao et al. [51] reported that implementing ISBAR during bedside handover in a rheumatology setting improved the completeness, accuracy, and satisfaction of the handover process, further supporting the positive role of structured interventions in audit-related outcomes.

### Magnitude of educational impact measured by effect size

Cohen’s d analysis demonstrated very large effect sizes in the knowledge and ISBAR domains, particularly among head nurses for knowledge retention (d = 22.61) and among staff nurses for the ISBAR and audit scores (d = 9.0). These values far exceed the thresholds commonly reported in the literature on clinical education, indicating an exceptionally effective intervention. Overall, these findings suggest that bedside handover training had a highly significant and lasting impact on participants’ knowledge and compliance behaviors. The magnitude of the effect sizes indicates a robust and meaningful change rather than a minor or moderate improvement, underscoring the effectiveness and sustainability of the training program. This finding is consistent with Sapri et al. [52], who reported average educational effect sizes in nursing studies ranging from 0.8–1.5. The dramatic improvements observed in the current study emphasize the training’s quality and contextual relevance. Moreover, the large effect sizes and durable outcomes align with evidence suggesting that structured, behavior-informed implementation strategies can promote high fidelity and long-term adoption in clinical practice (Curtis et al. [53]). Additionally, Jeong et al. [54] reported standardized mean differences of up to SMD = 1.55 in evidence-based practice (EBP) education among undergraduate nursing students. Beyond the clinical setting, educational research shows that average effect sizes for impactful learning interventions typically hover below 0.5, making your observed effects d = 9–22 astoundingly large by comparison. For instance, Evans and Yuan [55] reported that the 90th percentile effect size across a broad range of randomized educational studies was only approximately 0.45 standard deviations.

The findings of this study underscore the critical importance of integrating structured bedside handover training into nursing education and leadership development programs. The significant and sustained improvements observed in knowledge, ISBAR compliance, and documentation practices highlight the value of empowering nursing staff at all levels with effective communication tools. The incorporation of simulation-based learning and reflective feedback further facilitates the translation of theoretical knowledge into clinical practice, enhancing patient safety and care continuity. Given the demonstrated benefits across ICU settings and the potential applicability to other clinical units, healthcare institutions should prioritize ongoing bedside handover education as a core component of quality improvement initiatives. Future research should explore tailored strategies to address generational learning differences and evaluate long-term outcomes to sustain and expand these positive effects.

### Implications of the study

The findings of the study suggest that structured bedside handover training can significantly increase nurses’ knowledge and compliance with standardized communication protocols, such as ISBAR. These improvements can directly influence hospital policies focused on patient safety and care continuity. Healthcare institutions can integrate regular bedside handover training sessions into ongoing nursing education programs to promote consistent, high-quality information exchange. Policies should also emphasize the importance of involving patients in the handover process, fostering transparency and patient-centered care. Furthermore, nursing leaders can use these results to advocate for unit-level accountability and standardized handover practices as part of quality improvement initiatives. By embedding structured communication training into hospital policy, organizations can strengthen their safety culture, minimize errors, and improve overall patient outcomes.

## Conclusion of the study

In conclusion, this study reinforces the critical role of structured bedside handover training in enhancing nurses’ knowledge, ISBAR compliance, and overall quality of clinical handover practices. These findings contribute to the growing body of evidence that supports bedside handover as a vital component of patient safety and effective communication in healthcare settings. Improved knowledge and adherence to structured frameworks such as ISBAR not only streamline information exchange but also foster accountability, reduce errors, and promote continuity of care. These outcomes highlight the importance of embedding handover training into institutional policies and nursing education programs. Sustained improvements will depend on continuous training, leadership support, and regular audits to monitor compliance and identify areas for refinement. Ultimately, bedside handover, when effectively implemented, can serve as a key strategy in advancing safe, patient centered care.

On the basis of these findings, bedside handover training should be integrated into mandatory hospital orientation programs for all newly appointed nurses. Additionally, periodic refresher courses and simulation-based workshops should be established to sustain improvements in knowledge and practice.

## Limitations of the study

The study was conducted in a single public hospital’s intensive care units, which may limit the generalizability of the findings to other hospital settings or specialties. Additionally, the absence of a control group limits the ability to draw causal inferences. Without a comparison group, it is difficult to rule out alternative explanations for the observed improvements, such as external influences or natural progression over time. This represents a significant constraint on the internal validity of the findings. Additionally, the follow-up period was relatively short (three months), it allowed for timely assessment of early implementation outcomes, which can be considered a strength in understanding immediate impacts. Future research should employ a more rigorous experimental design, such as randomized controlled trials, and incorporate longer follow-up periods to assess the sustainability of change over time.

## Data Availability

Owing to confidentiality concerns, the data and materials used in the current research cannot be made publicly available. However, they are available from the corresponding author upon reasonable request.

## Abbreviations

ICU: Intensive care unit
ISBAR: “Identification” “Situation, “Background “Assessment” “Recommendation
IPSG: International Patient Safety Goals
